# Prevalence of *Filifactor alocis* and Its RTX Protein-Encoding Gene, *ftxA*, Among Periodontitis Patients in Sweden

**DOI:** 10.3390/pathogens15070662

**Published:** 2026-06-23

**Authors:** Rolf Claesson, Jessica Radu, Zeinab Razooqi, Anders Johansson, Jan Oscarsson

**Affiliations:** Department of Odontology, Umeå University, 901 87 Umeå, Sweden; rlkc1952@gmail.com (R.C.); jessicaradu@me.com (J.R.); zeinab.razooqi@umu.se (Z.R.)

**Keywords:** *F. alocis*, *ftxA*, periodontitis, subgingival plaque, co-colonization, Sweden

## Abstract

The oral pathogen *Filifactor alocis* encodes a repeats-in-toxin (RTX) protein, FtxA, that is encoded by the *ftxA* gene; it is present in approximately 50% of known isolated strains from various infected oral sites, including periodontitis, peri-implantitis, and root canal infections. It has been determined from PCR assessment of periodontally diseased cohorts in Ghana and Australia. Based on current knowledge, *ftxA* appears to be associated with both the progress and severity of periodontitis. This finding could potentially be linked to enhanced levels of *ftxA*-positive *F. alocis*, relative to *ftxA*-negative strain, and/or, in addition, a synergy between *ftxA*-positive strains and other periodontal pathogens. The exact mechanism remains unclear but may depend on an FtxA-mediated shifting of the host cell response toward immunosuppression. The main objective of the present work was to evaluate the prevalence and loads of *F. alocis* and the presence of *ftxA* in subgingival plaque in patients recruited for periodontal treatment in Sweden. This observational study included all samples that were received from external clinics over one full year (n = 71 patients). Our findings revealed that *F. alocis* was carried by 49 (69%) of the individuals, with the prevalence of *ftxA* amounting to 42.9% (n = 21). In 32 of the 71 samples, *F. alocis* could be quantitatively assessed. In this sub-population of *F. alocis*-positive patients, high loads of the bacterium were not related to age, and high loads were more frequently observed upon carriage of *ftxA*. The presence of, and co-colonization with, *F. alocis* with four additional periodontal pathogens was also evaluated. *F. alocis* was notable in that it co-colonized with all of the other species. Moreover, it was detected alongside two and even three of the other species within the same sample.

## 1. Introduction

Periodontitis is a globally prevalent disease that degrades the tooth-supporting tissues via induced interactions from accumulating growth of microbial communities (dental plaque) toward host immunity [1]. While hitherto essentially associated with Gram-negative bacterial species such as *Porphyromonas gingivalis* and *Aggregatibacter actinomycetemcomitans*, a Gram-positive anaerobe, *Filifactor alocis,* is now recognized as an emerging key immune modulator in periodontitis [2,3], peri-implantitis [4], and endodontic infections [5]. Both the prevalence and load of *F. alocis* increase in severe cases of periodontitis [6].

A synergistic relationship appears to exist between the prevalence of *F. alocis* and *A. actinomycetemcomitans* in periodontitis [7]. In some studies, the simultaneous presence of *F. alocis* and *A. actinomycetemcomitans* was observed to enhance *F. alocis* levels, although this correlation depended on the particular *F. alocis* strain with which it interacted [7,8,9,10]. *A. actinomycetemcomitans* may subsequently be outcompeted by the strictly anaerobic *F. alocis* in deeper periodontal pockets [11,12]. Indeed, this pattern primarily occurs at sites with deep periodontal pockets, aligning with increased tooth attachment loss [6]. Concomitantly, assessment of a population in southern India revealed increased levels of *F. alocis* in subgingival plaque collected from patients with chronic periodontitis and lower levels in those with an aggressive form of this disease [13]. In a recent study, *F. alocis* was demonstrated to promote a shift from a homeostatic oral microbial community toward a dysbiotic state [14]. The exact mechanism remains unclear; however, it requires the presence of Toll-Like Receptor 2 (TLR2).

The virulence of *F. alocis*, as hitherto known, includes its potential to manipulate neutrophils to extend their lifespan [15,16], potentially promoting bacterial survival by preventing the formation of neutrophil extracellular traps (NETs) [15]. This process may subsequently block phagosome maturation, reducing reactive oxygen species (ROS) production and subsequently inhibiting neutrophil-mediated bacterial clearance. These outcomes may allow *F. alocis* to proliferate in the oxidative-stress-rich environment of the periodontal pocket. Potential virulence factors of *F. alocis* that promote this process include superoxide reductase (FA796) and hypothetical protein FA51, in the ROS detoxification pathway, suggesting roles in ROS resistance [17]. In addition, *F. alocis* can delay apoptosis of macrophages by prolonging their inflammatory cascade, hence promoting periodontitis [18]. Additionally, evidence suggests that a complement-inhibitory protein produced by *F. alocis*, FACIN, is involved in arginine metabolism [19].

*Filifactor* Toxin A (FtxA) is a recently recognized Repeats-in-Toxins (RTX) protein encoded and produced in 50% of known *F. alocis* strains isolated [20,21]. Of particular note, *ftxA* is associated with both the progress and severity of periodontitis [9,10], which may be linked to enhanced loads of *F. alocis* [9] and/or synergy with *A. actinomycetemcomitans*. One plausible mechanism underlying this association was demonstrated in a recent study involving THP-1 macrophage-like cells, i.e., that FtxA shifts the host cell response toward immunosuppression [22]. As the hitherto main cohorts assessed regarding the prevalence and levels of *F. alocis* and the prevalence of *ftxA* have been one in Ghana and one in Australia, respectively, we aimed to investigate this phenomenon among a population of patients recruited for periodontological treatment in Sweden. We performed an observational study, compiling data from all routine samples received from external clinics (n = 71 patients undergoing periodontal treatment) during one full year. Prevalence and levels of *F. alocis* are reported, in addition to the presence of *ftxA* and the percentage (%) of total viable count (TVC) for *F. alocis* and for a selection of additional periodontal bacterial pathogens.

## 2. Materials and Methods

### 2.1. Research Subjects

The present study was performed at the Dental School, Umeå University, Sweden, and the samples included all of those received from external clinics over the course of one full year (2016; n = 71 patients undergoing periodontal treatment). The collected samples were assessed essentially as described in [23]. The individuals were sampled for subgingival plaque using paper points [24] (30 s sampling time), which were thereafter transported in VMGAIII medium [25] from the external clinics. The samples were subsequently microbiologically analyzed at the Clinical Oral Microbiology Laboratory of the Dental School for routine periodontal diagnostic purposes. We emphasize that this study is a summary of data obtained from analyses of these clinical samples sent to the clinical laboratory for identification and characterization. No sample was taken solely for research purposes, and the data cannot be traced to any of the sampled individuals. No data from the patients other than the microbial analysis were included.

### 2.2. Bacterial Cultivation from the Subgingival Plaque Samples

Samples from periodontal pockets of patients have been routinely analyzed for the presence of periodontitis-associated bacterial species at the Clinical Laboratory of Oral Microbiology, Dental School, in Umeå, Sweden, for more than 35 years according to established methods. In brief, after transportation of the samples in VMGAIII medium [25] to the clinical laboratory, the samples were processed as described, including laboratory-based identification of the periodontitis-associated species *A. actinomycetemcomitans*, *P. gingivalis*, *Parvimonas micra*, and *Prevotella intermedia/nigrescens* [23,26,27]. Proportions of these organisms in the samples were calculated by respectively comparing concentrations of the individual bacterial species with the total number of bacteria (TVC).

### 2.3. DNA Isolation

In parallel, samples collected in VMGAIII medium underwent DNA isolation using a GXT NA Extraction Kit^®^ (Hain Lifescience, GmBH, Nehren, Germany) and an Arrow automated extraction instrument (Liaison IXT, DiaSorin AB., Solna, Sweden). The exact procedure was described in a previous study [12]. The amount of total extracted DNA was quantified using a NanoDrop (Thermo Fisher Scientific, Uppsala, Sweden) instrument.

### 2.4. PCR Analysis of F. alocis and ftxA Prevalence

The presence or absence of *F. alocis* in the samples was assessed using isolated DNA as a template and previously validated oligonucleotide primers targeting the *F. alocis* 16S rRNA gene, as described in [28]. The prevalence of *ftxA* in the samples was determined using a forward (5′-GGCTCAGATACCTACTTCTTC-3′) and a reverse (5′-GAAGGCTATGATTTGATTGTTTCC-3′) oligonucleotide primer, which amplify a 798-base pair (bp) internal fragment of the *ftxA* gene, as described previously [12,20].

### 2.5. Quantitative PCR Analysis of F. alocis

Quantification of *F. alocis* loads as cells/mL of the sample, based on the isolated DNA, was performed as previously described [10], using a Corbett Research Rotor-Gene 6000 Rotary Analyze instrument (QIAGEN, Valencia, CA, USA) and oligonucleotide primers targeting the *F. alocis* 16s rRNA gene [28]. To calculate the proportion of *F. alocis* in the samples, qPCR data (cells/mL) were compared with the TVC of the sample, determined as described above (2.2). A high *F. alocis* load was arbitrarily set as ≥10,000 cells/sample, and low loads as <10,000 cells/sample, as previously described [10].

### 2.6. Statistical Analysis

Significant differences between sample groups were examined using Student’s *t*-test [29]. The odds ratio (OR) was calculated with the MedCalc calculator (https://www.medcalc.org/en/calc/odds_ratio.php; accessed during 30 April 2026), and *p*-values ≤ 0.05 were indicative of significant differences. Based on the limited number of samples in the present study, no further statistical analysis methods were used. This factor represents a limitation of this observational study, the main findings of which are related to the prevalence and loads of *F. alocis* and the presence of *ftxA* in periodontitis patients in Sweden.

### 2.7. Ethical Considerations

All procedures were conducted in accordance with the guidelines of the local ethics committee at the Medical Faculty of Umeå University, which are in accordance with the Declaration of Helsinki (75th WMA General Assembly, Helsinki, October 2024).

## 3. Results

### 3.1. Prevalence of F. alocis and ftxA in the Sampled Population

**Figure 1:**
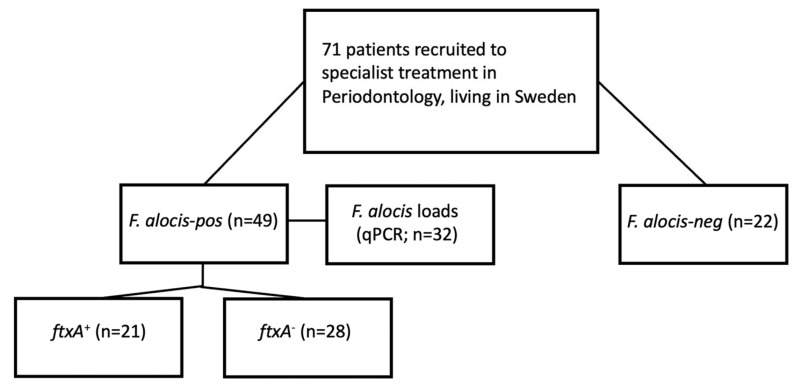
Flowchart illustrating a schematic outline of the present study regarding the assessment of *F. alocis* and *ftxA*. This is an observational study based entirely on routine samples received during a one-year period and analyzed at the Clinical Oral Microbiology Laboratory of the Dental School. Subgingival plaque samples from 71 patients were initially assessed by means of DNA-isolation and subsequent PCR analysis to determine the presence or absence of *F. alocis*. The *F. alocis*-positive samples (n = 49) were further analyzed to determine the loads of this bacterium (qPCR) and the presence or absence of *ftxA*.

**Figure 2:**
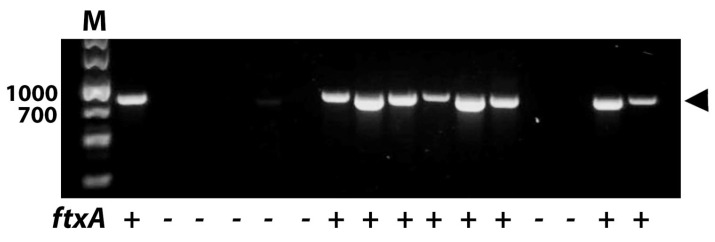
Determination of the presence or absence of *ftxA* in the 49 samples positive for *F. alocis* by means of PCR using oligonucleotide primers specific for *ftxA*, amplifying a 798 bp internal fragment of this gene (arrow). The readout as *ftxA*-positive or -negative is indicated below each lane. Sizes (bp) of selected bands in the DNA molecular weight marker (M) are indicated. The figure illustrates a representative PCR experiment from screening a selection of the 49 samples.

A flowchart summarizing the outline of the present study regarding the assessment of *F. alocis* and *ftxA* is shown in Figure 1. The readouts of all parameters assessed in the present work for each of the 71 samples are shown in Supplementary Table S1. Based on the PCR-assessment of DNA extracted from the subgingival plaque of the 71 patients, 49 (69.0%) individuals were identified as *F. alocis* carriers and the remaining 22 (31.0%) as non-carriers. The 49 *F. alocis*-positive samples were subsequently analyzed to determine the prevalence of *ftxA* using PCR (Figure 2), revealing that this gene was present in 21 (42.9%) and absent in the remaining 28 (57.1%).

### 3.2. Prevalence and Sample Loads of F. alocis in Relation to Carriership of ftxA

**Figure 3:**
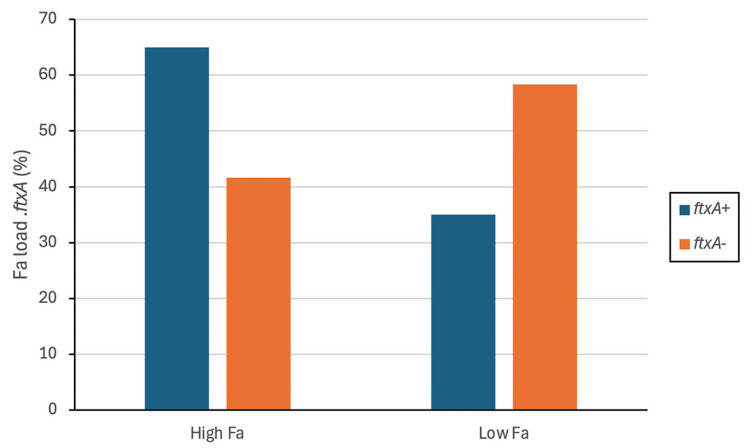
Prevalence and sample load of *F. alocis* (Fa) in relation to carriership of *ftxA*. Proportion of *F. alocis* sample load in relation to *ftxA* presence (+/−). High Fa load ≥10,000 cells/sample (n = 18); low Fa load <10,000 cells/sample (n = 14).

As schematically indicated in Figure 1, the concentrations of *F. alocis* were determined by means of qPCR in 32 of the samples positive for this organism, and the proportion of *F. alocis* relative to the TVC was simultaneously calculated in these samples. Of the 32 samples, 20 were *ftxA*-positive (62.5%) and 12 *ftxA*-negative (37.5%). Of particular note, the number of samples with high loads of *F. alocis* (≥10,000 cells/mL) was higher in the *ftxA*-positive samples compared with the *ftxA*-negative samples (Figure 3) (OR for high-load Fa samples determined to be *ftxA*+ compared with low-load Fa samples = 2.600 [*p* = 0.2027]). Taken together, within the limitations of a small sample size, these results support the notion that carriers of *ftxA*-positive *F. alocis* exhibit higher loads of the bacterium.

### 3.3. Prevalence of Periodontitis-Associated Bacterial Species Among the 49 Samples Containing F. alocis

In the 71 samples, the prevalence and proportion of the TVC were also determined for the four additional periodontal pathogens, i.e., *A. actinomycetemcomitans*, *P. gingivalis*, *P. intermedia/nigrescens*, and *P. micra*. Our findings revealed their presence in 16–23 of the samples, and their proportions of the TVC were also assessed (Table 1; Supplementary Table S1).

### 3.4. Co-Colonization of Periodontal Pathogens Including F. alocis in the 71 Samples

To investigate potential synergism, co-colonization of the assessed bacterial species (*F. alocis* and the four additional periodontal pathogens), alone and in combination, was analyzed in all 71 samples (Table 2, Supplementary Table S1).

Our findings revealed apparent co-colonization in several combinations of up to four co-present periodontal pathogenic species in the same sample. Of particular note, *F. alocis*, alone among the tested species, was detected in 17 of the samples. *F. alocis* also exhibited a tendency to co-colonize with all four additional assessed species and was furthermore found together with two or three of the other species.

## 4. Discussion

The present observational study comprised all subgingival plaque samples collected with paper points from patients with periodontitis and sent from external clinics to the Clinical Oral Microbiology Laboratory of the Dental School for periodontal routine diagnostic purposes, over a one-year period (n = 71 patients). A major purpose of this work was to characterize the prevalence and carriage loads of the emerging oral pathogen *F. alocis* and the prevalence of its RTX-toxin-encoding gene, *ftxA*, in the specimens. To conduct our investigation, PCR and qPCR were used; in addition, co-colonization of four additional periodontitis-associated species was monitored. The number of periodontal species assessed may be considered limited; however, it represents the routine analyses performed, with the exception of *F. alocis* addition.

Although periodontitis is known as a complex multifactorial disease, involving a multi-species dysbiotic microbiota and numerous host factors, the present focus was mainly directed toward one bacterial species, *F. alocis*, and its ftxA gene. This approach was employed to enhance the understanding of the prevalence of *F. alocis* in Sweden and to assess whether the carriage was ftxA-positive or -negative among periodontitis patients.

*F. alocis* has attracted further interest in recent years following the discovery of genotypes that express FtxA, an RTX family protein that represents only the second identified RTX family member demonstrated to be produced by an oral bacterium, following the well-studied leukotoxin of *A. actinomycetemcomitans* [16,20,21,30]. Although both RTX proteins belong to the same protein family (the C-terminal part of FtxA shares 30% amino acid identity with Leukotoxin), they appear to behave differently toward host cells, with FtxA instead attenuating inflammation [22]. How FtxA physically interacts with the host remains unclear; however, the protein itself, in addition to its presence in extracellular vesicles, has recently been shown to exert an immunosuppressive effect on monocytic cells, attenuating both inflammation and apoptosis pathways [22]. The results of clinical studies have suggested a potential role of FtxA in the virulence of *F. alocis* in periodontal disease. In a longitudinal Ghanaian adolescent cohort, *ftxA*-positive *F. alocis* carriers exhibited higher bacterial loads and a greater prevalence of CAL progression [9,12]. The rationale for selecting a population with all individuals (n = 71) diagnosed with periodontitis was its suitability to enable further assessment of the role of *F. alocis* and its FtxA gene in periodontal disease by assessing a population in Sweden. The general prevalence of *F. alocis* in the population in Sweden has hitherto not been thoroughly investigated; however, a recent screening among adolescents in Västerbotten County (in which the authors assessed 61% of all adolescents born in 2001 and residing in Västerbotten) revealed that all (100%) periodontal cases carried *F. alocis*, in addition to 54% of the non-periodontal control cases [31]. In the above study, however, neither *F. alocis* levels nor *ftxA* prevalence were investigated.

In our studies on a Ghanaian adolescent cohort [9,12], our findings demonstrated the association between *ftxA* carriage and elevated *F. alocis* levels and periodontal disease progression, prompting us to suggest that *F. alocis*, and primarily *ftxA,* could serve as a biomarker for identifying younger individuals at higher risk of rapid progression, highlighting the potential value of *F. alocis* and *ftxA* in clinical diagnostics. The results from the present cross-sectional study are consistent with the hypothesis that *ftxA* may have such a role, as a clear majority of the *ftxA*-positive samples exhibited high loads of *F. alocis*, within the limitations of a small sample size, however.

A potentially synergistic behavior between *F. alocis* and *A. actinocytetemcomitans* was initially implicated based on observations using the Human oral microbe infection microarray (HOMIM), concluding that *A. actinomycetemcomitans*, *Streptococcus parasanguinis*, and *F. alocis* together may represent a consortium in the causation of localized aggressive periodontitis, by indicating sites of future bone loss [7]. This hypothesis has since been further supported by our group and others, analyzing the Ghanaian longitudinal adolescent cohort, reflecting a potential scenario in which these species co-present in the periodontal pocket, in which *A. actinomycetemcomitans* may be successively out-competed by *F. alocis* [9,11,12]. In our recent work, it was revealed that among individuals carrying *A. actinomycetemcomitans*, a majority harbored *ftxA*-positive *F. alocis*. This finding was supported by clinical readouts reflecting CAL, age group, and history of periodontitis [10]. In addition, this finding may reflect the hypothesis that effective co-existence of these two species requires *F. alocis* to produce FtxA, thereby enhancing its ability to survive within the periodontal pocket, at least among younger patients. Despite a generally low proportion (%) of TVC when present, *F. alocis* was still found alone in 17 of the 71 cases, in which co-carriage was assessed with four additional periodontitis-associated pathogens in combinations. The exact mechanism remains unknown. The observed co-colonization of *F. alocis* with the other periodontal pathogens assessed, including *A. actinomycetemcomitans*, is consistent with potential synergism and their association with periodontal disease pathogenesis [3].

## 5. Conclusions

Within the limitations of the small sample size, this observational study revealed that *F. alocis* was carried by 49 (69%) of the 71 assessed individuals, whereby 21 (42.9%) were *ftxA*-positive. In 32 of the 71 samples, the loads of *F. alocis* could be quantitatively assessed. In this sub-population of *F. alocis*-positive patients, a trend was observed whereby high loads of this bacterium were more frequently observed upon carriage of *ftxA*.

## Figures and Tables

**Table 1:** Prevalence of additional periodontitis-associated species in the 71 samples determined by means of Total Viable Counts (TVCs). *F. alocis* prevalence was estimated by means of conventional PCR and the proportion with quantitative PCR.

Bacterial Species	Identified	Prevalence (%)	Proportion of TVC (%)	
			Variation	Median
*F. alocis*	49	69	0.001–59 ^(1)^	0.27
*A. actinomycetemcomitans*	18	25	0.01–92	4.8
*P. gingivalis*	18	25	1.5–90	5.6
*P. micra*	23	23	0.6–38	8.4
*P. intermedia/nigrescens*	16	32	1.0–71	3.6

^(1)^ Although *F. alocis* was identified in 49 of the 71 samples, the proportion of this organism relative to TVC could be calculated in 32 samples.

**Table 2:** Co-colonization. Number of the 71 samples containing each of the assessed species when present alone and in combinations.

Bacterial Combination	Number of Cases
*F. alocis (Fa)*	17
*A. actinomycetemcomitans (Aa)*	4
*P. gingivalis (Pg)*	1
*P. micra (Pm)*	3
*P. intermedia/nigrescens (Pi/n)*	1
*Aa + Pg*	0
*Aa + Pm*	0
*Aa + Pi/n*	0
*Aa + Fa*	4
*Pg + Pm*	1
*Pg + Pi/n*	1
*Pg + Fa*	6
*Pm + Pi/n*	2
*Pm + Fa*	7
*Pi/n + Fa*	3
*Aa + Pg + Pm*	0
*Aa + Pg + Fa*	1
*Aa + Pm + Pi/n*	1
*Aa + Pm + Fa*	1
*Aa + Pi/n + Fa*	2
*Pg + Pm + Pi/n*	2
*Pg + Pm + Fa*	1
*Pg + Pi/n + Fa*	1
*Pm + Pi/n + Fa*	1
*Aa + Pg + Pm + Pi/n*	0
*Aa + Pg + Pm + Fa*	3
*Aa + Pm + Pi/n + Fa*	1
*Aa + Pg + Pi/n + Fa*	1
*Pg + Pm + Pi/n + Fa*	0
*Aa + Pg + Pm + Pi/n + Fa*	0
*Fa Aa Pg Pm Pi/n negative*	6

## Data Availability

The original contributions presented in this study are included in the article/Supplementary Material. Further inquiries can be directed to the corresponding authors.

## Supplementary Materials

The following supporting information can be downloaded at: https://www.mdpi.com/article/10.3390/pathogens15070662/s1, Table S1: The parameters determined regarding the individual 71 samples, respectively. Aa, *A. actinomycetemcomitans*, Pg, *P. gingivalis*, Pi/n *P. intermedia/nigrescens*, and Pm, *P. micra*. TVC, total viable count, and nd, not detected. Yellow indicates the 32 samples in which the loads of *F. alocis* could be determined by qPCR.
