# Supplementary material for: Prevalence of Filifactor alocis and Its RTX Protein-Encoding Gene, ftxA, Among Periodontitis Patients in Sweden
Source: Pathogens. 2026 Jun 23;15(7):662. doi: 10.3390/pathogens15070662 (PMC13415055; doi:10.3390/pathogens15070662)
Supplement: Supplementary file 1 [file pathogens-15-00662-s001.zip › pathogens-4331021-supplementary.pdf]

Supplementary Table 1. The parameters determined regarding the individual 71 samples, respectively. Aa, *A. actinomycetemcomitans* , Pg, *P. gingivalis* , Pi/n *P. intermedia/nigrescens* , and Pm, *P. micra* . TVC, total viable count, and nd, not detected.
Yellow indicates the 32 samples in which the loads of *F. alocis* could be determined by qPCR.

sample	*F. alocis*	*ftxA*	(cells/ml sample) of *F. alocis*	TVC (million cells/ml)	Aa (% of TVC)	Pg (% of TVC)	Pi/Pn (% of TVC)	Pm (% of TVC)
1	1		9442	16	nd	nd	3,4	nd
2	1	1	27	0,1	nd	nd	nd	33
3	1		198	4,8	nd	nd	nd	nd
4	1	1	2,2	0,1	nd	nd	nd	nd
5	1	1	560	32	nd	nd	24	nd
6	1	1	91	5,4	0,20	1,5	2,2	nd
7	1	1	637	45	nd	nd	0,6	nd
8	1	1	140	11	nd	nd	nd	5,1
9	1	1	19	2,4	18,0	nd	nd	18
10	1	1	154	21	nd	nd	11	1,9
11	1	1	71	10	nd	71	nd	1,8
12	1		4,7	0,7	0,60	nd	2,0	nd
13	1	1	132	35	0,70	nd	12	nd
14	1		47	14	nd	56	nd	nd
15	1	1	13	4,1	nd	nd	nd	nd
16	1	1	9,6	3	nd	nd	nd	nd
17	1	1	54	20	nd	nd	nd	2,4
18	1	1	55	21	4,0	2,0	nd	4,0
19	1		0,8	0,5	nd	nd	nd	nd
20	1		145	120	nd	nd	nd	nd
21	1		50	1,4	nd	nd	nd	nd
22	1	1	5,4	88	nd	nd	nd	3,9
23	1	1	13	11	0,08	13	nd	1,8
24	1	1	0,4	34	nd	63	nd	nd
25	1	1	0,05	1,2	nd	nd	nd	nd
26	1		3,6	0,2	nd	nd	nd	nd
27	1		0,2	26	nd	nd	nd	nd
28	1	1	0,18	2,2	nd	35	nd	nd
29	1		0,06	3,5	nd	nd	nd	nd
30	1		1,0	26	nd	nd	nd	nd
31	1	1	0,22	12	92	nd	nd	nd
32	1		0,16	20	nd	nd	nd	nd
33	1			0,2	79	nd	nd	nd
34	1			17	nd	nd	24	17
35	1			0,1	22,0	nd	nd	nd
36	1			21	0,01	nd	17	1,0
37	1			0,3	nd	nd	nd	nd
38	1			4,5	nd	12	8,5	nd
39	1			1,2	nd	nd	nd	71
40	1			1	nd	nd	nd	nd
41	1			0,1	nd	nd	nd	nd
42	1			0,1	nd	54	nd	32
43	1			4,9	nd	nd	nd	nd
44	1			74	nd	nd	nd	nd
45	1			5,9	nd	nd	nd	nd
46	1			57	nd	82	nd	nd
47	1			30	nd	nd	nd	nd
48	1			83	nd	90	nd	nd
49	1			29	nd	nd	nd	3,5
50		1		2,3	nd	nd	nd	18
51				11	0,004	26	nd	6,3
52				2,9	nd	nd	nd	nd
53				39	nd	62	nd	nd
54				0,09	4,8	nd	38	8,9
55				1,4	nd	nd	nd	nd
56				5,2	nd	nd	nd	19
57				5,1	6,6	nd	nd	nd
58				19	nd	87	3,6	nd
59				12	nd	nd	nd	nd
60				35	nd	60	nd	nd
61				34	8,8	nd	nd	nd
62				3,1	58	nd	nd	nd
63				1,1	nd	nd	nd	9,4
64				0,9	nd	nd	0,9	nd
65				15	nd	nd	1,3	2,6
66				0,8	nd	nd	nd	45
67				0,7	0,05	58	nd	nd
68				4,5	nd	19	11	11
69				0,3	53	nd	nd	nd
70				14	80	nd	nd	nd
71				7	nd	38	1,4	8,5
